# Pneumococcal Conjugate Vaccines and Otitis Media: An Appraisal of the Clinical Trials

**DOI:** 10.1155/2012/312935

**Published:** 2012-06-03

**Authors:** Mark A. Fletcher, Bernard Fritzell

**Affiliations:** Pfizer Specialty Care Business Unit-Vaccines, International Scientific & Clinical Affairs, Pfizer Inc., Cedex 14, 75668 Paris, France

## Abstract

*Streptococcus pneumoniae* is the predominant otitis media pathogen and its prevention through effective vaccination could diminish childhood illness and antibiotic use. This paper reviews 5 pneumococcal conjugate vaccine (PCV) trials that used otitis media as an endpoint: Northern California Kaiser Permanente (NCKP; vaccine, 7-valent PCV [PCV7]-CRM); Finnish Otitis Media (FinOM; vaccines, PCV7-CRM or PCV7-OMPC); Native American Trial (vaccine, PCV7-CRM); Pneumococcal Otitis Efficacy Trial (POET; vaccine, 11-valent PCV [PCV11]-PD). For the microbiological endpoint, vaccine efficacy against vaccine-serotype pneumococcal otitis media was about 60% across trials. Against the clinical endpoint of all episodes, vaccine efficacy was 7% (PCV7-CRM/NCKP), 6% (PCV7-CRM/FinOM), −1% (PCV7-OMPC/FinOM), and −0.4% (PCV7-CRM/Native American Trial); 34% against first episodes of ear, nose, and throat specialist-referral cases (PCV11-PD/POET). Both follow-up through 2 years of age, for the 5 trials, and long-term follow-up, for PCV7-CRM/NCKP and PCV7-CRM/FinOM, demonstrated greater vaccine efficacy against recurrent AOM and tympanostomy-tube placement, suggesting that vaccination against early episodes of AOM may prevent subsequent episodes of complicated otitis media. Although study designs varied by primary endpoint measured, age at follow-up, source of middle-ear fluid for culture, case ascertainment, and type of randomization, each clinical trial demonstrated vaccine efficacy against microbiological and/or clinical otitis media.

## 1. Introduction

Otitis media is commonly an acute infection of childhood that is most prevalent in children during their first years of life. Otitis media leads to substantial healthcare costs related to physician visits, antibiotic expenditures, and, for complicated cases, surgical procedures such as tympanostomy-tube placement. Otitis media is also a major factor in antibiotic use and the expansion of antibiotic resistance. Prior to the advent of an effective vaccine against the pneumococcal serotypes associated with otitis media, approximately two-thirds of children in the USA, for example, experienced at least 1 episode of otitis media, and almost 1 in 6 children experienced 3 or more episodes of otitis media in their first years of life. In vaccine clinical trials in the USA and Europe, where acute otitis media (AOM) was defined as the presence of a visibly abnormal tympanic membrane suggesting middle-ear effusion plus at least one sign of acute infection (e.g., fever), the rates of AOM episodes in control groups generally ranged from 1.2 to 1.8 per child per year (PCPY) in the first 2 years of life [1–4]. Identification of the source of infection by tympanocentesis confirms the diagnosis of AOM, but, outside of clinical trials, most AOM is diagnosed clinically based on signs and symptoms without confirmatory culture.

When there is microbiological confirmation that AOM is caused by bacteria, the principal isolates are *Streptococcus pneumoniae*, *Haemophilus influenzae*, or *Moraxella catarrhalis* with small contributions from *Staphylococcus aureus* or Group A *Streptococcus* [2, 5, 6]. The most commonly identified pathogen is *S. pneumoniae* which, prior to adoption of the 7-valent pneumococcal conjugate vaccine (PCV7), was isolated by myringotomy in approximately one-third to half of all cases [2, 5, 6]. Although more than 90 serotypes of *S. pneumoniae* have been identified [2], a limited number is associated with the majority of pneumococcal illness [7]. In a review of clinical data published between January 1970 and June 2008, the *S. pneumoniae* serotypes most frequently associated with AOM were 6A, 6B, 14, 19A, 19F, and 23F [8].

Three pneumococcal conjugate vaccine candidates were studied in clinical trials to assess efficacy against otitis media. The PCV7 vaccine targets the seven serotypes, 4, 6B, 9V, 14, 18C, 19F, and 23F, each individually conjugated to cross-reactive material (CRM197), the nontoxic diphtheria toxin analogue (PCV7-CRM; Pfizer [Philadelphia, PA, USA]) [2, 7]. An investigational vaccine containing the same seven *S. pneumoniae* serotypes as PCV7-CRM, but conjugated to a meningococcal outer-membrane protein complex (PCV7-OMPC; Merck [West Point, PA, USA]), also underwent clinical development, but it is currently not licensed [9]. Likewise, an investigational 11-valent PCV containing serotypes 1, 3, 4, 5, 6B, 7F, 9V, 14, 18C, 19F, and 23F, each conjugated to a cell-surface protein of *H. influenzae* (PCV11-PD; GlaxoSmithKline [Rixensart, Belgium]), underwent clinical development but was not submitted for licensure [4]. The seven serotypes 4, 6B, 9V, 14, 18C, 19F, and 23F, which are found in each formulation, account for 60% to 80% of the serotypes that commonly cause pneumococcal AOM, and also account for most of the antibiotic-resistant *S. pneumoniae* serotypes [2, 7, 14].

Five clinical trials tested these PCV candidates, and this paper reviews trial design and methods, the control group, efficacy endpoints, and results against otitis media as an endpoint. These trials differed in important aspects that will be considered, including the primary endpoint measured, age at follow-up, source of middle-ear fluid (MEF) for culture, case ascertainment, and type of randomization. As well as differences in methodology, the studies also differed by characteristics of the control group, including rate of otitis media reported from the control group, the underlying prevalence of *S. pneumoniae* AOM in the control group, and the proportion of vaccine-type AOM episodes in the control group. Clinical trial results will be recapitulated, both for the clinical trials and long-term follow-up.

## 2. Methods

The safety and efficacy of PCV7-CRM was investigated in two individually randomized, clinical trials—the Northern California Kaiser Permanente (NCKP) Vaccine Trial and the Finnish Otitis Media (FinOM) Vaccine Trial—and in a community-randomized clinical trial among Native American (Navajo and White Mountain Apache) children [2, 3, 10, 15]. PCV7-OMPC was investigated as an arm of the FinOM study [9]. PCV11-PD was studied in the individually randomized Pneumococcal Otitis Efficacy Trial (POET) [4].  Table 1 summarizes the key features of the trials [2–4, 9, 10, 12, 15].

Vaccine efficacy in the trials with otitis media as an outcome could be based on microbiological endpoints (pneumococcus or vaccine-serotype pneumococcus isolated from spontaneous drainage or MEF isolates) or clinical endpoints (e.g., episodes, recurrence, or risk of tympanostomy-tube placement). Clinical trials during the development of these vaccines found that their efficacies against a microbiological outcome, vaccine-serotype pneumococcus in MEF isolates, were quite similar (about 60%), yet vaccine efficacies against a clinical outcome, “clinical episodes” of otitis media, varied considerably (values ranging from −1 to 34%) [2, 4, 9]. With respect to clinical endpoint, the primary otitis media endpoint measured varied by study, as did the otitis media definition. For the NCKP Vaccine Trial, the primary outcome was number of episodes of or visits for otitis media, obtained from computerized records using diagnoses registered by emergency and pediatric physicians in the NCKP populations; for the FinOM Vaccine Trial, it was number of episodes of culture-confirmed, vaccine-serotype pneumococcal AOM (visibly abnormal tympanic membrane suggesting middle-ear effusion plus at least one sign of acute infection) obtained by the clinical investigators of the study; for the Native American Trial, it was visits with clinically diagnosed episodes of otitis media, including AOM, as documented by the patients' treating physicians; for POET it was first episode of vaccine-serotype pneumococcal AOM (abnormal tympanic membrane or presence of MEF, plus two predefined clinical symptoms within 14 days preceding the clinical diagnosis) confirmed by an ear, nose, and throat (ENT) specialist after referral by the patient's pediatrician.

### 2.1. NCKP Vaccine Trial

#### 2.1.1. NCKP Vaccine Trial Design and Methods

The NCKP Vaccine Trial was conducted in 23 medical centers in California, USA, from 1995 to 1998. Children were randomly assigned to receive PCV7-CRM or control (meningococcal serogroup C conjugate) vaccine, administered at ages 2, 4, 6, and 12 to 15 months; they were followed until approximately 2 years of age and then until 3.5 years of age (long-term follow-up, see 3.5.1., NCKP Vaccine Trial) [3, 15]. The study enrolled a total of 37,868 children, 18,927 received 1 or more doses of PCV7-CRM [15]. Otitis media was based on diagnoses made by emergency physicians or pediatricians in the NCKP-healthcare network [15]. A microbiological endpoint was obtained from the 23 cases of spontaneously draining ruptured tympanic membranes with culture of a vaccine-serotype pneumococcus (intent-to-treat group, 17 from children in the control group and 6 from children in the PCV7-CRM group; fully vaccinated group, 12 from children in the control group and 4 from children in the PCV7-CRM group).

#### 2.1.2. NCKP Vaccine Trial Control Group

The study control group included 18,942 children who received at least 1 dose of control vaccine [3]. In the control group, there were 18,286 physician/clinic visits for otitis media among those aged younger than 1 year, 21,721 visits among those aged 1 to 2 years, and 6446 visits among those aged older than 2 years [3]. Children aged younger than 1 year had a rate of 2.65 otitis media visits/year compared with 2.01 otitis media visits/year for children aged 1 to 2 years and 1.18 otitis media visits/year for children aged older than 2 years [3]. The rate of otitis media visits peaked at 10 months of age [3]. For children aged 8 to 12 months, this translated to a rate of 22 to 24 visits per 100 children per month. For children aged 12 to 30 months, the rate was 11 to 12 visits per 100 children per month [3].

#### 2.1.3. NCKP Vaccine Trial Efficacy Endpoints

The primary-efficacy endpoint of the NCKP Vaccine Trial was the efficacy of PCV7-CRM in preventing invasive pneumococcal disease (IPD) due to the pneumococcal serotypes included in the vaccine. Prevention of clinical episodes of otitis media (i.e., number of otitis media visits, including AOM visits) was a secondary endpoint [15].

### 2.2. FinOM Vaccine Trial

#### 2.2.1. FinOM Vaccine Trial Design and Methods

In the FinOM study, eight clinics were set up in three Finnish cities between 1995 and 1999 [2, 9]. Families in these cities were informed of the study through various public media, and parents interested in participating presented to the clinics for enrollment. Healthy infants aged 2 months received study (PCV7-CRM or PCV7-OMPC) or control (hepatitis B virus) vaccine as a 3-dose primary series at 2, 4, and 6 months and a booster at 12 months [2]. (In FinOM, 55% of the children born in the study area were enrolled.) Children who were vaccinated with PCV7-OMPC received it on the same schedule as PCV7-CRM, but with the following exception: after 3 doses of PCV7-OMPC, about a fifth of study vaccine children (*n* = 187) received, as the final 12-month dose, a 23-valent pneumococcal polysaccharide vaccine (Pneumovax 23 [PPSV23]; Merck [West Point, PA, USA]) [9].

During the study period, parents were encouraged to bring their children to a participating clinic if symptoms of respiratory illness or AOM developed. Trained medical staff performed myringotomy with aspiration of MEF from all children presenting with any clinical symptom suggesting AOM to establish bacterial culture and pneumococcal serotyping; MEF cultures were obtained during 93% of AOM clinic visits [2]. New episodes of AOM were defined as those occurring more than 30 days after a previous episode or at any time after a previous episode if a different serotype or pathogen was identified [2]. The FinOM study design followed all AOM episodes because, as the authors noted, recurrent AOM is common [1, 5, 6]. In addition, all AOM episodes were included in the analyses because selection of a single event per child would likely have resulted, again as noted, in biased population estimates due to the complicated interdependence of recurrence and age [16]. Vaccine efficacy from clinical and microbiological endpoints was evaluated in study children aged 6.5 to 24 months [2]. Most children who participated in the FinOM study were included in the long-term FinOM Trial Follow-up (see 3.5.2., FinOM Vaccine Trial) [13].

#### 2.2.2. FinOM Vaccine Trial Control Group

In the FinOM control group, there were 1345 AOM episodes in 831 children [2, 9], and 587 (71%) children experienced 1 or more documented AOM-related event(s) during the follow-up period, with a range of 1 to 15 events per child (median: 3 events) [2, 9, 16]. The overall incidence was 1.24 episodes of AOM PCPY [2]. Among the 1345 episodes, MEF was obtained from 1267 episodes, of which 1082 were culture-confirmed bacterial episodes [2]. The success rate of obtaining a MEF sample was 97.2% when middle-ear effusion was suspected and an attempt was made to collect MEF by myringotomy [2]. At least 1 MEF sample was available from each of the 1819 AOM events (among 2595 ears of patients with AOM, as an episode could be bilateral) [16]. *S. pneumoniae*, as the only pathogen or in a mixed culture, represented 44% of all bacterial AOM in the control group (age range: 2 to 24 months [16]); 60% of pneumococcal AOM was caused by serotypes included in the vaccine and 20% was caused by vaccine-related serotypes [2].

#### 2.2.3. FinOM Vaccine Trial Efficacy Endpoints

The primary-efficacy endpoint of the FinOM Vaccine Trial was the total number of episodes of AOM due to vaccine serotypes [2]. Upon examination by the clinic physician, AOM in FinOM was defined as the abnormal appearance of the tympanic membrane suggesting effusion, plus a single feature of acute clinical illness such as fever, earache, diarrhea, or signs of respiratory infection [2]. Secondary-efficacy endpoints included vaccine efficacy in preventing first and subsequent episodes of AOM, and efficacy against recurrent AOM [2].

### 2.3. Native American Trial

#### 2.3.1. Native American Trial Design and Methods

A double-blind, community-randomized clinical trial conducted among Navajo and White Mountain Apache children from April 1997 to August 2000 compared the efficacy of PCV7-CRM for pneumococcal disease against a control (meningococcal group C conjugate) vaccine [12, 17]. Infants were scheduled to receive 3 doses of vaccine at 2, 4, and 6 months of age, followed by a booster administered at 12 to 15 months of age [10]. This study used community randomization; 8292 children were enrolled, selected from 38 geographically defined, residence-based randomization units that were clusters of existing, well-defined communities, and 8045 of these children were included in the group-randomized design of this efficacy trial and received vaccine (PCV7-CRM = 4142; control = 3903) [10, 13, 17]. At trial completion, 4476 children were eligible for a retrospective chart review based on having been aged less than 7 months when they received the first dose of vaccine, having received 3 doses of study vaccine by age 12 months, and having had a minimum of 30 days between doses [12]. From this group of 4476 children, a sample of 1000 charts was considered necessary to provide statistical power; charts from 944 children were chosen randomly [12]. Researchers evaluated “clinically diagnosed episodes of otitis media (all types of otitis media diagnosis)” occurring in study children until they reached age 2 years [12]. The primary efficacy subgroup (*n* = 856) met the chart-review criteria (PCV7-CRM = 424; control = 432); the per-protocol subgroup (*n* = 803) was first vaccinated on or after 42 days of age, third dose was received before 365 days of age, booster dose was received between 365 and 480 days of age, and there was at least a 60-day interval between the end of the primary and the booster dose. Efficacy in the per-protocol group was measured from 14 days after completion of the third dose, while it began immediately after the first dose in the primary efficacy group. As a microbiological endpoint, there were 51 spontaneously draining otitis media episodes that grew *S. pneumoniae* from the efficacy trial population (*n* = 8292). From the 51 spontaneously draining otitis media episodes that grew *S. pneumoniae*, serotype data was obtained from 23 isolates collected for serotyping (11 PCV7 serotypes and 12 non-PCV7 serotypes); only the PCV7 serotype results are available [12].

#### 2.3.2. Native American Trial Control Group

In the primary efficacy subgroup of children who met chart-review criteria (*n* = 856), there was a high burden of disease; in the per-protocol subgroup of children who met criteria for age-appropriate vaccination (*n* = 803), there were 736 episodes of AOM (816 otitis media episodes) in the control group (*n* = 432), an incidence of 1.4 episodes PCPY: for all otitis media of 1.5 episodes PCPY, and for AOM of 1.3 episodes PCPY [12].

#### 2.3.3. Native American Trial Efficacy Endpoints

The endpoints of the Native American Trial included efficacy of PCV7-CRM in preventing IPD, as well as indirect effects of the vaccine in a community through reduction in carriage and secondary attack rates due to the pneumococcal serotypes included in the vaccine [10]. The primary efficacy endpoint used in the subanalysis that evaluated PCV7-CRM otitis media efficacy was prevention of clinically diagnosed episodes of otitis media, including AOM [12].

### 2.4. POET

#### 2.4.1. POET Design and Methods

POET was conducted in pediatric centers in the Czech Republic and Slovakia from 2000 to 2004, and healthy infants aged approximately 3 months received 4 doses (a 3-dose primary series at 3, 4, and 5 months plus a booster at 12 to 15 months) with PCV11-PD or control (hepatitis A virus) vaccine [4]. (In regions participating in POET, up to 20% of the infant population was enrolled [18].) Parents of these infants would bring their sick children to local pediatricians, who referred children with suspected AOM to ENT specialists who also were participating in the study. The ENT specialist performed tympanocentesis for bacterial culture and serotyping after confirming the local pediatrician's diagnosis [4]. Follow-up for the per-protocol group started 2 weeks after the third vaccine dose and continued until the age of 24 to 27 months [4]. New episodes of AOM were defined as those occurring more than 30 days after a previous episode or at any time after a previous episode if a different serotype or pathogen was identified [4]. This trial focused on microbiological endpoints in the first two years of life, and no long-term follow-up of study participants was conducted.

#### 2.4.2. POET Control Group

In the POET control group, 499 clinical episodes of AOM (i.e., specialist-referral episodes) were recorded in 2452 children who completed the study per-protocol. Of these clinical episodes, 61% (306/499) were culture-confirmed bacterial episodes [4]. Of the 306 confirmed bacterial episodes, 62% (189/306) were culture-confirmed pneumococcal episodes; 75% (141/189) of culture-confirmed pneumococcal episodes were caused by serotypes included in the vaccine, and 12% (23/189) were caused by vaccine-related serotypes [4]. The overall incidence of AOM was 0.12 episodes of AOM PCPY (i.e., 12.5 episodes per 100 person-years of follow-up) [4].

#### 2.4.3. POET Efficacy Endpoints

In POET, the primary endpoint was efficacy against the first episode of AOM due to vaccine pneumococcal serotypes [4]. Upon examination by a pediatrician and then an ENT specialist, AOM in POET was based upon an abnormal tympanic membrane or the presence of MEF, plus two predefined clinical symptoms within 14 days preceding the diagnosis. The main secondary-efficacy endpoint of POET was efficacy against the first episode of AOM caused by nontypable *H. influenzae* [4].

## 3. Results

The clinical trial results are summarized in Tables 2-6 [2–4, 7, 9, 12, 13, 15]. Per-protocol and intent-to-treat efficacy values, where available, are reported in Table 2 (e.g., otitis media episodes, recurrent otitis media, spontaneous perforation, and risk of tympanostomy-tube placement) [2–4, 9, 12, 15]. The number of microbiological isolates due to vaccine serotypes in the control group or the pneumococcal vaccine group is presented in Table 3 [2, 4, 9, 12]. In the long-term follow-up (PCV7-CRM/NCKP and PCV7-CRM/FinOM), vaccine per-protocol efficacy against clinical endpoints (e.g., visits or episodes, recurrence, tympanostomy-tube placement, AOM-related ambulatory visits, or rate of antibiotic prescriptions for AOM), and intent-to-treat values where available, are in Table 4 [3, 7, 13]. The vaccine-attributable reduction values (otitis media episodes, bacteria in MEF isolates, pneumococcus in MEF isolates, and vaccine-serotype pneumococcus in MEF isolates), as calculated, are reported in Table 5 [2, 4, 9, 12]. The vaccine efficacy and vaccine-attributable reduction values (i.e., number of cases prevented per 100 children vaccinated), by clinical trial, are presented in Table 6 [2, 4, 9, 12].

### 3.1. NCKP Vaccine Trial

The PCV7-CRM group included 18,927 children who received at least 1 dose of PCV7-CRM [15]. In this study, efficacy against vaccine serotypes was 66.7% in the per-protocol analysis of the subsample cultured after spontaneous rupture (Tables 2 and 3) [15]. Vaccine efficacy in preventing the number of otitis media visits, including AOM visits, was 7.0% (95% confidence interval [CI] = 4.1–9.7) in the per-protocol analysis [3, 15]. Other efficacy results are shown in Table 2 [3, 15].

### 3.2. FinOM Vaccine Trial

#### 3.2.1. PCV7-CRM

In the FinOM PCV7-CRM group, 1251 episodes of AOM were diagnosed among 831 vaccinated children during the 18-month follow-up period [2]. Among the 1251 episodes, 1177 were confirmed by the presence of MEF, of which 965 were culture-confirmed bacterial episodes (Table 5) [2]. Efficacy in preventing total number of episodes of pneumococcal AOM due to vaccine serotypes (obtained by myringotomy), the primary endpoint, was 57% (95% CI = 44–67) in the per-protocol analysis (Tables 2 and 3) [2]. The overall incidence of AOM was 1.16 episodes PCPY (versus 1.24 in the control group), for a vaccine efficacy against the number of episodes of AOM (first and subsequent episodes) of 6% (95% CI = −4 to 16) [2]. Other efficacy results are shown in Table 2 [2].

#### 3.2.2. PCV7-OMPC

In the PCV7-OMPC arm of FinOM, 1364 episodes of AOM were diagnosed in 835 vaccinated children, of which 1279 were confirmed through the presence of MEF, with 1073 culture-confirmed bacterial episodes (Table 5) [9]. As described earlier (see Section 2.2.1), some of the children in the PCV7-OPMC study arm received a PPSV23 vaccine as the final dose at 12 months after receiving 3 primary series doses of PCV7-OMPC at 2, 4, and 6 months of age [9]. PCV7-OMPC efficacy in preventing AOM due to vaccine serotypes (obtained by myringotomy), the primary endpoint, in the per-protocol analysis was 56% (95% CI = 44–66) (Tables 2 and 3) [9]. Vaccine efficacy in preventing all episodes of AOM was –1% (95% CI = −12 to 10) [9]. When these groups were split out, the overall incidence of AOM caused by vaccine serotypes was 7.55 episodes per 100 person-years among children who received 4 doses of PCV7-OMPC and 7.66 episodes per 100 person-years among children who received 3 doses of PCV7-OMPC plus a PPSV23 final dose [9]. Vaccine efficacy against AOM due to vaccine serotypes in the per-protocol groups was 60% (95% CI = 43–72) in the 4-dose PCV7-OMPC group (*n* = 631) and 65% (95% CI = 34–81) in the PPSV23 final dose group (*n* = 187) (Table 2) [9].

### 3.3. Native American Trial

Of the 856 children in the primary-efficacy analysis, 424 had received PCV7-CRM [13]. The PCV7-CRM-vaccinated children had a total of 1092 AOM episodes during the study period, giving an incidence of 1.4 AOM episodes PCPY (Table 1) [12]. In the per-protocol analysis, there were 706 AOM episodes (785 otitis media episodes) in the PCV7-CRM group (Table 5). Vaccine efficacy was 64% (95% CI = −34 to 90) against otitis media due to vaccine serotypes, as measured in a subgroup of 23 isolates from spontaneously draining otitis media episodes that had been collected for serotyping (Tables 2 and 3) [12]. Vaccine efficacy against clinically diagnosed episodes of otitis media, including AOM (i.e., “otitis media, acute otitis media, bilateral otitis media, chronic otitis media, otitis media with perforation, otorrhea, pressure-equalizing tube placement, perforated tympanic membrane, serous otitis media, and bullous myringitis”) was 0% (95% CI = −19 to 16) [12]. Other available efficacy results are shown in Table 2 [12].

### 3.4. POET

In the PCV11-PD group, 333 clinical episodes of ENT-referred AOM were diagnosed in 2455 children who were vaccinated, of which 322 were episodes with MEF obtained by aspiration, and 178 were culture-confirmed bacterial episodes [4]. Of the 178 confirmed bacterial episodes, 92 were culture-confirmed pneumococcal episodes (Table 5) [4]. PCV11-PD vaccine efficacy against first occurrence of AOM due to vaccine serotypes in the per-protocol analysis, the primary endpoint, was 52.6% (95% CI = 35.0 to 65.5) (Tables 2 and 3) [4]. PCV11-PD vaccine efficacy against first occurrence of AOM due to nontypable *H. influenzae* (NTHi) was 31.1% (95% CI = −3.7 to 54.2), the main secondary endpoint [4].

Vaccine efficacy against any episode of ENT-referred AOM during per-protocol follow-up was 34% (95% CI = 21 to 45) [4]. (The overall incidence of AOM was 0.083 episodes PCPY in the vaccine group versus 0.125 AOM episodes PCPY in the control group [4].) Vaccine efficacy against all episodes of clinical AOM (ENT-referred and episodes that were not referred to an ENT) was apparently not reported.

### 3.5. Trial Results: Long-Term Follow-up

Long-term follow-up information is available from the PCV7-CRM/NCKP and the PCV7-CRM/FinOM clinical trials and is summarized in Table 4. Where available, results for per-protocol and intent-to-treat analyses are given in the table.

#### 3.5.1. NCKP Vaccine Trial

The follow-up to the NCKP Vaccine Trial, conducted from 1998 to 1999, looked specifically at otitis media endpoints (e.g., overall incidence, risk of frequent otitis media, or use of tympanostomy tubes) in children aged 3.5 years or younger [3]. Children received a 4-dose schedule (2, 4, 6, and 12 to 15 months), with per-protocol analysis beginning 14 days after completion of the primary series. PCV7-CRM reduced otitis media visits by 7.8% (95% CI = 5.4–10.2) (Table 4) [3]. The reductions in otitis media visits in the PCV7-CRM group versus control, based on age, were 8.2% (95% CI = 5.1–11.1) for children younger than 1 year, 8.7% (95% CI = 5.8–11.6) for children aged 1 to 2 years, and 3.7% (95% CI = −1.8 to 8.8) for children older than 2 years [3].

#### 3.5.2. FinOM Vaccine Trial

In the FinOM Trial Follow-up, families of children who had been monitored until the age of 24 months in FinOM and who were still living in the area were contacted and invited to a follow-up visit when the children were aged 4 to 5 years (Table 4) [13]. The primary analysis population comprised 756 children (403 from the PCV7-CRM group and 353 from the control group) [13]. Information on tympanostomy-tube placement for the primary analysis was obtained through collective review from medical centers of private patient records from children in FinOM (up to age 24 months), parent interviews at the follow-up visit, and district hospital records [13]. A secondary analysis included 1490 children and used data from hospital records up to June 2001 in the study area [13]. Per-protocol vaccine efficacy for all tympanostomy-tube placement was 39% (95% CI = 4–61) in the primary-analysis population and 44% (95% CI = 19–62) in the secondary-analysis population (Table 4) [13].

## 4. Discussion and Conclusions

These clinical trials demonstrate that AOM is a vaccine-preventable disease; the impact of PCVs on microbiological endpoints (pneumococcus or vaccine-serotype pneumococcus), clinical endpoints, or both has been documented in five studies that differed in many respects, for example, primary endpoint measured, age at follow-up, source of MEF for culture, case ascertainment, and type of randomization [2–4, 12, 15]. The primary endpoint differed between PCV11-PD/POET, defined as the first episode of AOM [4], and the other 4 studies, where all episodes or all visits for AOM were used [2, 3, 10, 12, 15]. The age at follow-up differed as well. In PCV7-OMPC/FinOM, PCV7-CRM/Native American Trial, and PCV11-PD/POET, children were followed until the second year of life [3, 4, 9, 10, 12], whereas children from PCV7-CRM/NCKP and PCV7-CRM/FinOM were followed, respectively, until the age of 3.5 years or until 4 to 5 years [13]. Investigators in PCV7-CRM/FinOM, PCV7-OMPC/FinOM, and PCV11-PD/POET obtained MEF by myringotomy with aspiration from children presenting with the otitis media endpoint who matched the myringotomy criteria [2, 4, 9]. For PCV7-CRM/FinOM, this included all children presenting with AOM; for PCV7-OMPC/FinOM, this involved all children presenting with respiratory infection or symptoms suggesting AOM if AOM was diagnosed at the visit; for PCV11-PD/POET, pediatricians decided whether to refer children with AOM clinical features to an ENT specialist for confirmation and myringotomy (Table 1). By contrast, MEF was obtained only from cases with spontaneous rupture in the PCV7-CRM/NCKP and PCV7-CRM/Native American trials [3, 10, 12]. Case ascertainment included all otitis media events for 4 of the trials (PCV7-CRM/NCKP, PCV7-CRM/FinOM, PCV7-OMPC/FinOM, and PCV7-CRM/Native American) [2, 3, 9, 10, 12]. In PCV11-PD/POET, ENT referral was not systematic and may have been reserved for more severe cases. Subsequent to publication of the results, the PCV11-PD/POET principal investigator noted that POET was not designed to capture every AOM episode, but only the most “disturbing” cases that led to ENT referral [1]. A recent meta-analysis of conjugate-vaccine trials noted that vaccines have a greater impact on the most severe forms of AOM [19]. In a reanalysis of the PCV7-CRM/FinOM dataset that applied PCV11-PD/POET case definitions, the efficacy of PCV7-CRM against clinical AOM or against vaccine-serotype pneumococcal AOM was not affected significantly by differences in case definitions. In emphasizing the completeness of case ascertainment in PCV7-CRM/FinOM, the authors of the reanalysis concluded that the reported differences in vaccine-efficacy values between trials were due to results of several factors, including differences in study methodology, particularly case detection, as well as variations in the epidemiology of pneumococcal or serotype-specific pneumococcal AOM, or the effect of PCV11-PD against NTHi [20]. Finally, four of the studies (PCV7-CRM/NCKP, PCV7-CRM/FinOM, PCV7-OMPC/FinOM, and PCV11-PD/POET) used individual randomization whereas the PCV7-CRM/Native American Trial used community-based randomization.

For the microbiological endpoint, the vaccine efficacy against pneumococcal vaccine serotype otitis media was similar across the following trials: 67%, PCV7-CRM/NCKP; 57%, PCV7-CRM/FinOM; 64%, PCV7-CRM/Native American Trial; 56%, PCV7-OMPC/FinOM; 58%, PCV11-PD/POET (Tables 2 and 3) [2–4, 12, 15]. By contrast, the values for the clinical endpoints varied between trials. Against all clinical episodes, vaccine efficacy was 7% (PCV7-CRM/NCKP), 6% (PCV7-CRM/FinOM), −1% (PCV7-OMPC/FinOM), and −0.4% (PCV7-CRM/Native American Trial); it was 34% against first episodes of ENT specialist-referral cases (PCV11-PD/POET). Both for follow-up through 2 years of age for the five trials, and in long-term follow-up for two of the trials (PCV7-CRM/NCKP, until 3.5 years of age; PCV7-CRM/FinOM, until 4–5 years of age), larger values for vaccine efficacy in preventing recurrent AOM and reducing the need for tympanostomy tubes were demonstrated, suggesting that prevention of early episodes of AOM through vaccination may prevent subsequent episodes of complicated otitis media [3].

An intriguing finding was the lack of vaccine efficacy against clinically diagnosed episodes of otitis media, including AOM, in the PCV7-CRM/Native American Trial, while the vaccine-attributable reduction against otitis media in the PCV7-CRM/Native American Trial was 7 cases avoided per 100 children vaccinated, which is the same as the value demonstrated for PCV11-PD/POET but less than the value for PCV7-CRM/FinOM (12 cases avoided per 100 children vaccinated) (Table 5). Furthermore, PCV7-CRM showed good efficacy otherwise against the microbiological endpoint, pneumococcal vaccine serotype otitis media, comparable to each of the other four clinical trials (Table 2) [10, 12]. This lack of vaccine efficacy against clinically diagnosed episodes of otitis media may have been due to a lower proportion of vaccine serotypes in the study population, where only about half of all IPD was caused by vaccine serotypes, which is a low proportion of vaccine serotype IPD compared with other populations [10, 12]. In the AOM subgroup, vaccine serotypes were present in 11 cultures (8 in the control group and 3 in the PCV7-CRM group) from 23 cultures overall of MEF obtained from spontaneously draining cases, which is consistent with the IPD findings, but because systematic MEF cultures were not performed, this remains speculation [12]. The lack of vaccine efficacy demonstrated against clinical otitis media also could be related to the use of community, rather than individual, randomization. This suggests that, interfering with transmission, the basis of the “herd” effect that has been associated with PCV7-CRM vaccine, may play an important role in vaccine efficacy against all-cause otitis media [17]. There also was no clinical efficacy demonstrated in the PCV7-OMPC/FinOM trial, which used individual, randomization, although it is not possible to differentiate the impact on any AOM between children who received 4 doses of PCV7-OMPC and those who received 3 doses of PCV7-OMPC plus a PPSV23 final dose because separate vaccine efficacy calculations were not provided.

In addition to the differences in methodology, the studies also differed in the characteristics of the rate of otitis media reported from the control group, the underlying prevalence of *S. pneumoniae* AOM in the control group, and the proportion of vaccine-type AOM episodes in the control group. The overall incidence of AOM in the PCV11-PD/POET control group was low, approximately a tenth that is observed in FinOM and other studies [20]. Whether this was due to the POET study design, which may not have reflected the full burden of AOM, or factors unique to the location or time of the study were involved (e.g., antibiotic use, family size, or daycare-center population) is unclear. The authors of a reanalysis of the FinOM dataset that applied POET case definitions highlighted that [20] “The AOM episodes captured may have been a subgroup of the total number of cases.” The incidence of AOM PCPY in the control groups of PCV7-CRM/NCKP (2.0 to 2.6) and the PCV7-CRM/Native American (1.4) trials are more consistent with the FinOM control group (1.24) than the PCV11-PD/POET control group (0.12) (Table 1) [2, 3, 7, 10, 12, 15]. A difference in the underlying prevalence of *S. pneumoniae* AOM in the control groups of FinOM (38% of positive cultures) and POET (62% of positive cultures) may be relevant to the observed difference in vaccine efficacy against episodes of otitis media [19]. The proportion of vaccine-serotype AOM episodes was higher in the PCV11-PD/POET study (28% of clinical AOM episodes in the POET study control group compared with 19% in the FinOM study control group), which may be due to true epidemiologic differences or may reflect differences in case detection [20]. In the PCV7-CRM/NCKP and PCV7-CRM/Native American trials, by contrast, microbiological samples were obtained only after spontaneous rupture. Microbiological findings from these 2 studies cannot help cast any light on the issue because they present the number of vaccine-serotype pneumococcus only, distributed between the PCV7-CRM and the control group.

Based on the vaccine-attributable reductions documented during both FinOM trials, the PCV7-CRM/Native American Trial, and the PCV11-PD/POET, the predicted impact of a national immunization program with these vaccines on otitis media episodes, bacteria in MEF isolates, pneumococcus in MEF isolates, and vaccine-serotype pneumococcus in MEF isolates can be estimated (Tables 5 and 6). The PCV7-CRM/FinOM trial can provide a good basis for estimating the potential impact of pneumococcal vaccination. Adherence throughout the study was high (95%), rates of AOM in FinOM were consistent with those reported in most other studies, and nearly all (93%) AOM cases were confirmed through myringotomy [2, 20]. During a 1.5-year period (December 1995 to April 1997), more than half (55%) of the children born in the study area were enrolled, and these children were followed until the age of 24 months. Based on this double-blind, randomized, controlled design, intended to measure the direct effect of the vaccine [2], the anticipated impact of vaccination with PCV7-CRM is prevention of approximately 12 AOM visits per year for every 100 children younger than 2 years of age who are vaccinated (Tables 5 and 6). This has been realized in numerous surveillance studies in the USA, where between 23 and 25 AOM visits per year were prevented for every 100 children (less than 2 years of age) vaccinated [21, 22] (e.g., using data reported by Zhou et al. [21], it can be calculated that 929 visits per 1000 person-years were prevented during the period 2001 to 2004; divided by 4 years, this yields 232.5 visits per 1000 person-years prevented annually, or 23 visits avoided per year per 100 children vaccinated). The reduction in nasopharyngeal carriage of vaccine-serotype pneumococci among vaccinated infants and the consequential reduction in transmission [23] established from a PCV7-CRM national immunization program [22] may account, at least in part, for the fact that postlicensure surveillance endpoint values are even greater than could have been anticipated from FinOM. PCV7 uptake nationwide was approximately 41% in 2002, 68% in 2003, and 73% in 2004, when the US study was conducted [24]. Other possible factors on the impact of PCV7-CRM in the US national immunization program include the long-term positive impact on subsequent episodes of AOM resulting from the prevention of an initial case of pneumococcal otitis media in a young child [16], as well as any changes in the management of otitis media that may have happened since the widespread introduction of PCV7-CRM, variations in the severity of seasonal influenza, or enhanced uptake of trivalent-inactivated influenza vaccine among infants [21, 25, 26].

Clinical experience with PCV7-CRM since its widespread adoption following initial licensure in 2000 demonstrates reduced rates of all clinical episodes of AOM and reduced rates of AOM-related outpatient visits and hospitalizations. Changes in the burden of otitis media after availability of PCV7-CRM vaccination include 36.4% vaccine efficacy against hospitalization for AOM in Italy (regional immunization program, 2 + 1 schedule) [27], 38% reduction in rates of otorrhea per emergency department visit in Greece (national immunization program, 3 + 1 schedule) [28], a 36% reduction in emergency visits due to suspected AOM in children at risk for recurrent AOM (rAOM) in Sweden (randomized, controlled trial, 3 + 1 schedule) [29], and, as noted, a 42.7% reduction in the rate of ambulatory visits attributable to AOM in USA (national immunization program, 3 + 1 schedule) [21]. In PCV11-PD/POET, vaccine efficacy against first-episode, ENT-referred, and MEF-positive AOM caused by nontypable *H. influenzae*, a secondary endpoint of the study, was 35.3% [4, 30]. PCV11-PD is conjugated to a cell-surface protein of *H. influenzae* [30], whereas the other vaccines were conjugated to CRM197, the nontoxic diphtheria toxin analogue (PCV7-CRM), or to OMPC, the meningococcal outer-membrane protein complex (PCV7-OMPC). Although direct protection against NTHi might have added to PVC11-PD overall vaccine efficacy in POET [20], protection against NTHi AOM in populations vaccinated uniquely with PCV7-CRM has been demonstrated. Data compiled from the emergency department visit-based study in Greece conducted in an 8-year period in children aged 0 to 14 years with AOM events complicated by otorrhea (i.e., MEF cultures from spontaneous draining AOM) found that there were statistically significant declines in the rates of otorrhea due to *H. influenzae* (−20%), as well as pneumococcal otorrhea (−48%), and overall visits (−38%) [28]. With respect to the randomized controlled trial in Sweden [29], in addition to the reduction in emergency visits, there were also statistically significant reductions in AOM episodes (−38%) and ventilation tube insertions (−50%) in children. This study had a particular design as it was a clinical trial of children identified to already be at high risk of rAOM because of a first episode before the age of 6 months, who were then vaccinated appropriately according to age with a follow-up period that began 4 weeks after the first vaccination (at approximately 8 months of age) and continued until the child was 2 years of age; therefore, the results of AOM episodes, emergency department visits, or ventilation tube insertions among young children with a high risk for recurrent AOM may be different for AOM among generally healthy infants, for example.

These improvements demonstrated that the availability of PCV7-CRM vaccination has crucial implications for morbidity from childhood illness and for the direct and indirect costs of medical care. In the USA, PCV7-CRM vaccination of infants and young children has resulted in a calculated 32% decrease in average annual spending related to AOM visits between a prevaccination (1997 to 1999) and a post-vaccination (2001 to 2004) period, savings of almost a half billion US dollars each year [21]. Compared with the years 1997 to 1999 (before vaccination became routine), antibiotic prescriptions related to AOM decreased by 42% in 2004 [21]. Approximately 2 years after widespread introduction of PCV7-CRM in the NCKP-healthcare system, for instance, the percentage of penicillin-resistant (minimum-inhibitory concentration ≥2 *μ*g/mL) pneumococcal isolates among all ages decreased from a high of 15% in 2000 to 5% in mid-2003 [31]. Judicious use of antibiotics could be expected to maintain these gains.

PCV7-CRM was first approved in 2000 in USA (Prevnar; Pfizer [Philadelphia, PA, USA]) for active immunization in infants and children against IPD and pneumococcal otitis media caused by the serotypes in the vaccine, and it was approved in 2001 in the European Union (Prevenar) for active immunization against vaccine-serotype IPD in infants and children, subsequently also encompassing noninvasive pneumococcal infections, pneumonia and otitis media, caused by the serotypes targeted by the vaccine [11, 32]. The PCV11-PD studied in POET was a prototype vaccine that was not submitted for licensure, but a 10-valent vaccine (PCV10), based on PCV11-PD and covering the same serotypes as PCV7-CRM plus serotypes 1, 5, and 7F, uses a mixed conjugate protein technology and was first licensed in the European Union in 2009 (Synflorix; GlaxoSmithKline [Rixensart, Belgium]) for active immunization against vaccine-serotype IPD and otitis media in infants and children caused by the serotypes targeted by the vaccine [30]. A 13-valent vaccine (PCV13-CRM) approved in 2010 in the USA (Prevnar 13, Pfizer) and in the European Union (Prevenar 13, Pfizer) for active immunization in infants and children against IPD, pneumococcal pneumonia, and pneumococcal otitis media caused by the serotypes in the vaccine, which covers serotypes included in PCV7-CRM plus serotypes 1, 3, 5, 6A, 7F, and 19A [33]. To date, no otitis media-efficacy studies or post-introduction surveillance otitis media effectiveness studies with the PCV10 or PCV13-CRM have been published.

In conclusion, otitis media is a common childhood disease with potentially serious consequences for those children afflicted with recurrent or severe manifestations. Its prevention holds the potential to produce widespread reductions in morbidity, antibiotic use (including the associated acquisition of resistance), and healthcare expenditures. Although the PCV7-CRM vaccine appears to have had an impact on otitis media morbidity since its introduction into national immunization programs (such as in the USA [21, 22, 24–26], Italy [27], or Greece [28]), otitis media continues to be an issue due to changing microbiology and other factors. If the newer vaccines, PCV10 or PCV13-CRM, demonstrate a further impact in clinical trials or in post-introduction surveillance against microbiologically confirmed otitis media (e.g., pneumococcal otitis media, vaccine serotype-pneumococcal otitis media, or otitis media due to other otopathogens), clinical otitis media (e.g., otitis media episodes, recurrent otitis media, or risk of tympanostomy-tube placement), or both, they will be valuable additions in the effort to prevent this widespread childhood disease.

## Figures and Tables

**Table 1:** PCV clinical trials with otitis media as an endpoint [2–4, 9, 10, 12, 15].

	NCKP [3, 15]	FinOM [2]	FinOM [9]	Native American Trial [10, 12]	POET [4]
Study vaccine	PCV7-CRM	PCV7-CRM	PCV7-OMPC	PCV7-CRM	PCV11-PD

Period	1995–1998	1995–1999	1995–1999	1997–2000	2000–2004

No. of children	37,868	1662	1666	8045	4907

No. in vaccine and control	18, 926 vaccine, 18, 942 control	831 vaccine, 831 control	835 vaccine, 831 control	4142 vaccine, 3903 control	2455 vaccine, 2452 control

Enrollment	Healthy 2-month-old infants	Healthy 2-month-old infants	Healthy 2-month-old infants	Healthy infants aged 6 weeks to 24 months*	Healthy infants aged 6 weeks to 5 months

Study period	Children aged 2 months to 3.5 years	Children aged 2 months to 4-5 years	Children aged 2 months to 2 years	Children aged 6 weeks to 24 months	Children aged 6 weeks to 24–27 months

Incidence PCPY, control group	2.0–2.6 visits	—	—	—	—
	—	1.24 episodes	1.24 episodes	1.4 episodes	0.12 episodes

Vaccination schedule	2, 4, 6, and 12–15 months	2, 4, 6, and 12 months	2, 4, 6, and 12 months^†^	2, 4, 6, and 12–15 months	3, 4, 5, and 12–15 months

Design	Prospective, individually randomized (1:1), double-blind, controlled	Prospective, individually randomized (1:1), double-blind, controlled	Prospective, individually randomized (1:1), double-blind, controlled	Prospective, community randomized, double-blind, controlled	Prospective, individually randomized (1:1), double-blind, controlled

Control vaccine	Meningococcal serogroup C conjugate	Hepatitis B virus	Hepatitis B virus	Meningococcal serogroup C conjugate	Hepatitis A virus

Primary otitis media endpoint	Number of episodes of or visits for otitis media	Number of episodes of AOM due to vaccine pneumococcal serotypes	All episodes of culture-confirmed pneumococcal AOM caused by vaccine serotypes	Clinically diagnosed episodes of otitis media, including AOM	First episode of AOM due to vaccine pneumococcal serotypes

Otitis media definition	Obtained from computerized records using diagnoses registered by emergency and pediatric physicians in the NCKP populations	Visibly abnormal tympanic membrane (color, position, or mobility) suggesting middle-ear effusion plus at least one sign of acute infection^‡^	Visibly abnormal tympanic membrane (color, position, or mobility) suggesting middle-ear effusion plus at least one sign of acute infection^‡^	Otitis media visits, as documented by the patients' treating physicians, were recorded	Abnormal tympanic membrane (redness, bulging, or loss of light reflex) or presence of MEF (simple or pneumatic otoscopy or microscopy), plus two predefined clinical symptoms within 14 days preceding the clinical diagnosis^§^
Myringotomy criteria	Not routinely obtained	All children presenting with AOM	All children presenting with respiratory infection or symptoms suggesting AOM if AOM was diagnosed at the visit	Not performed	Pediatricians decided whether to refer children with AOM clinical features to an ENT specialist for confirmation and myringotomy

Source of MEF	Cultures of spontaneously ruptured tympanic membranes	MEF sample from myringotomy with aspiration for bacterial culture and pneumococcal serotyping	MEF sample from myringotomy with aspiration for bacterial culture and pneumococcal serotyping	Samples obtained from spontaneously draining otitis media episodes for bacterial culture and serotyping	MEF sample from myringotomy with aspiration for bacterial culture and pneumococcal serotyping

*The primary efficacy cohort included children aged 6 weeks to less than 7 months at enrollment who received the full vaccine schedule.

^†^187 children in the vaccine group received a 23-valent pneumococcal polysaccharide vaccine at the fourth (booster) dose, instead of PCV7-OMPC.

^‡^Fever, earache, irritability, diarrhea, vomiting, acute otorrhea (not caused by otitis externa), and other symptoms of respiratory infection.

^§^Ear pain, ear discharge, fever, irritability, hearing loss, vomiting, diarrhea, lethargy, or anorexia.

ENT: ear, nose, and throat.

PCV7-CRM: pneumococcal conjugate vaccine candidate targeting serotypes, 4, 6B, 9V, 14, 18C, 19F, and 23F, each individually conjugated to cross-reactive material (CRM197), the nontoxic diphtheria toxin analogue; PCV7-OMPC: pneumococcal conjugate vaccine candidate targeting serotypes, 4, 6B, 9V, 14, 18C, 19F, and 23F, each individually conjugated to a meningococcal outer-membrane protein complex; PCV11-PD: pneumococcal conjugate vaccine candidate targeting serotypes 1, 3, 4, 5, 6B, 7F, 9V, 14, 18C, 19F, and 23F, each conjugated to a cell-surface protein of *H. influenzae*.

**Table 2:** Vaccine efficacy values in the PCV clinical trials with otitis media as an endpoint [2–4, 9, 12, 15]. Adapted with permission from Fletcher and Fritzell, 2007 Elsevier Ltd. All rights reserved [7].

		NCKP [3, 15]	FinOM [2]	FinOM [9]	Native American Trial [12]	POET [4]
Study vaccine		PCV7-CRM	PCV7-CRM	PCV7-OMPC	PCV7-CRM	PCV11-PD
Period		1995–1998	1995–1999	1995–1999	1997–2000	2000–2004
Country		USA	Finland	Finland	USA	Czech Republic, Slovakia
No. of children		37,868	1662	1666	856	4907
Age		2–24 months	2–24 months	2–24 months	6 weeks to 24 months	6 weeks to 24–27 months

Clinical endpoints*: vaccine efficacy, % (95% CI)						

Otitis media episodes		7 (4–10)	6 (−4 to 16)	−1 (−12 to 10)	0 (−19 to 16) −3 (−21 to 12)**	34 (21–45)
Recurrent otitis media†						
3/4		9 (3–15)	16 (−6 to 35)	—	5 (−52 to 41) 14 (−34 to 44)**	56 (−2 to 81)
4/5		12 (2–21)	—	—	—	—
5/6		23 (7–36)	—	—	—	—
Spontaneous perforation		—	23 (−18 to 50)	—	—	—
Risk of first tympanostomy-tube placement		20 (2–35)	4 (−19 to 23)	—	28 (−225 to 84) 22 (−256 to 83)**	60 (−27 to 88)

S*. pneumoniae* microbiological endpoints*: vaccine efficacy, % (95% CI)						

All serotypes		—	34 (21–45)	25 (11–37)	—	52 (37–63)
Vaccine serotypes	PP	67 (—)	57 (44–67)	56 (44–66) 60 (43–72)^‡^ 65 (34–81)^§^	64 (−34 to 90)	58 (41–69)
	ITT	65 (—)	54 (41–64)	—	—	53 (35–66)
Cross-reactive serotypes		—	51 (27–67)	−5 (−47 to 25) −21 (−98 to 17)^‡^ −25 (−88 to 49)^§^	—	66 (22–88)
Any other serotypes		—	−33 (−80 to 1)	−27 (−70 to 6) −22 (−86 to 19)^‡^ −146 (−405 to 20)^§^	—	8 (−64 to 49)

*See Table 1 for primary otitis media endpoint, otitis media definition, myringotomy criteria, and source of MEF in each study.

**Primary efficacy group (see Section 2.3.1. Native American Trial Design and Methods).

^†^Number of episodes in 6 months/number of episodes in 1 year.

^‡^Among children who received PCV7-OMPC booster, during a follow-up period lasting from 12 to 24 months.

^§^ Among children who received a final 12-month dose of 23-valent pneumococcal polysaccharide vaccine (Pneumovax 23 [PPSV23], Merck [West Point, PA, USA]) [9], during a follow-up period lasting from 12 to 24 months.

Reported values are rounded to whole numbers; dash line indicates not reported.

CI: confidence interval; ITT: intent to treat; PP: per-protocol.

**Table 3:** Case split (control group  : pneumococcal vaccine group) for *S. pneumoniae* serotypes in the PCV clinical trials with otitis media as an endpoint [2, 4, 9, 12].

	Case split (control vaccine group : pneumococcal vaccine group)			
	FinOM [2]	FinOM [9]	Native American Trial [12]*	POET [4]

Study vaccine	PCV7-CRM	PCV7-OMPC	PCV7-CRM	PCV11-PD
Serotype				
1	—	—	—	1 : 1
3	13 : 13	13 : 11	—	17 : 20
4	4 : 2	4 : 1	2 : 0	3 : 0
5	—	—	—	0 : 0
6B	56 : 9	56 : 12	0 : 0	24 : 3
7F	—	—	—	1 : 0
9V	11 : 5	11 : 2	0 : 0	8 : 3
14	26 : 8	26 : 11	1 : 0	22 : 1
18C	17 : 7	17 : 8	2 : 0	5 : 3
19F	58 : 43	58 : 37	3 : 3	43 : 24
23F	82 : 33	82 : 40	0 : 0	18 : 5

*Data obtained from 23 isolates collected for serotyping (11 PCV7 serotypes and 12 non-PCV7 serotypes) from 51 spontaneously draining otitis media episodes that grew *S. pneumoniae*; only the PCV7 serotype results are available.

For PCV7-CRM/NCKP, there were 16 cases of spontaneously draining ruptured tympanic membranes with culture of a vaccine-serotype pneumococcus in the fully vaccinated group (i.e., child <16 months of age who received three or more doses of vaccine, and child ≥16 months old who had received four doses of vaccine), 12 from children in the control group, and 4 from children in the PCV7-CRM group children. Four cases in fully PCV7-CRM-vaccinated children were serotype 19F; distribution of vaccine-serotype isolates in the control group was not reported [3, 15].

Dash line indicates not reported.

**Table 4:** Vaccine efficacy values in the long-term follow-up of PCV clinical trials with otitis media as an endpoint [3, 13]. Adapted with permission from Fletcher and Fritzell, 2007 Elsevier Ltd. All rights reserved [7].

Follow-up clinical trials			
		NCKP [3]	FinOM [13]
Study vaccine		PCV7-CRM	PCV7-CRM
Period		1998–1999	1999–2001
No. of children		27,754	756
Age		Until aged 3.5 years	Until aged 4–5 years

Clinical endpoints*: vaccine efficacy, % (95% CI)			

Otitis media visits (NCKP) or episodes (FinOM)	PP	8 (5–11)	8 (−2 to 16)
	ITT	7 (5–9)	—
Recurrent otitis media**			
3/4		10 (7–13)	18 (1–32)
5/6		—	50 (15–71)^†^
≥10^‡^		26 (12–38)	—
All tympanostomy-tube placements		23 (−10 to 46)	39 (4–61)^†,§^
			44 (19–62)^†,¶^
Rate of AOM-related ambulatory visits		8 (5–10)	—
Rate of antibiotic prescriptions for AOM		6 (4–7)	—

*See Table 1 for primary otitis media endpoint, otitis media definition, myringotomy criteria, and source of MEF in each study.

**Number of episodes in 6 months/number of episodes in 1 year.

^†^In children diagnosed with “chronic otitis media with effusion.”

^‡^Efficacy against 10 or more episodes within 6 months.

^§^ Primary analysis set (see Section 3.5.2. FinOM Vaccine Trial).

^¶^ Secondary analysis set (see Section 3.5.2. FinOM Vaccine Trial).

Reported values are rounded to whole numbers; dash line indicates not reported.

CI: confidence interval; ITT: intent to treat; PP: per-protocol.

**Table 5:** Vaccine-attributable reduction values based on otitis media episodes and MEF isolates (bacteria, pneumococcus, or vaccine-serotype pneumococcus) from the PCV clinical trials with otitis media as an endpoint (PP groups) [2, 4, 9, 12].

Otitis media endpoint	No. of cases (no. vaccinated)		Difference*	Vaccine-attributable reduction^†^
	Vaccine group	Control group		
Otitis media episodes				
PCV7-CRM/FinOM	1251 (786)	1345 (794)	−94	12
PCV7-OMPC/FinOM	1364 (805)	1345 (794)	+19	NA
PCV7-CRM/Native American	785 (424)	816 (432)	−31	7
PCV11-PD/POET	333 (2455)	499 (2452)	−166	7
Bacteria in MEF isolates^‡^				
PCV7-CRM/FinOM	965 (786)	1082 (794)	−117	15
PCV7-OMPC/FinOM	1073 (805)	1082 (794)	−9	1
PCV7-CRM/Native American	—	—	—	—
PCV11-PD/POET	178 (2455)	306 (2452)	−128	5
Pneumococcus in MEF isolates				
PCV7-CRM/FinOM	271 (786)	414 (794)	−143	18
PCV7-OMPC/FinOM	314 (805)	414 (794)	−100	12
PCV7-CRM/Native American	—	—	—	—
PCV11-PD/POET	92 (2455)	189 (2452)	−97	4
Vaccine-serotype pneumococcus in MEF isolates				
PCV7-CRM/FinOM	107 (786)	250 (794)	−143	18
PCV7-OMPC/FinOM	110 (805)	250 (794)	−140	17
PCV7-CRM/Native American	3 (424)	8 (432)	−5	1
PCV11-PD/POET	60 (2455)	141 (2452)	−81	3

*Vaccine group minus control group.

^†^Vaccine-attributable reduction: no. of cases prevented per 100 children vaccinated, which is the difference (vaccine group minus control group) divided by the no. vaccinated, then normalized per 100 children vaccinated.

^‡^ *S. pneumoniae*, *H. influenzae*, and *M. catarrhalis*, or any combination of these.

Dash line indicates not reported.

NA: not applicable. PP: per-protocol.

**Table 6:** Vaccine efficacy and vaccine-attributable reduction values in the PCV clinical trials with otitis media as an endpoint [2, 4, 9, 12].

PCV7-CRM/FinOM [2]	Otitis media episodes		Bacteria*	Sp	Vaccine serotype
Vaccine efficacy	7%		—	34%	57%
Vaccine-attributable reduction^†^	12 per 100		17 per 100	18 per 100	18 per 100
				
PCV7-OMPC/FinOM [9]	Otitis media episodes		Bacteria*	Sp	Vaccine serotype
Vaccine efficacy	−1%		—	25%	56%
Vaccine-attributable reduction^†^	—		1 per 100	12 per 100	17 per 100
				
PCV7-CRM/Native American Trial [12]	Otitis media episodes		Bacteria*	Sp	Vaccine serotype
Vaccine efficacy	−0.1%		—	—	64%
Vaccine-attributable reduction^†^	7 per 100		—	—	1 per 100
					
PCV11-PD/POET [4]	Outpatient visit episodes	Specialist referral episodes	Bacteria*	Sp	Vaccine serotype
Vaccine efficacy	—	32%	42%	52%	58%
Vaccine-attributable reduction^†^	—	7 per 100	5 per 100	4 per 100	3 per 100

*Culture-confirmed *S. pneumoniae*, *H. influenzae*, *M. catarrhalis*, or any combination of these.

^†^Vaccine-attributable reduction: no. of cases prevented per 100 children vaccinated, which is the difference (vaccine group minus control group) divided by the no. vaccinated, then normalized per 100 children vaccinated (see Table 5 for calculations).

Dash line indicates not reported.

Bacteria: bacteria in MEF isolates; Sp: pneumococcus in MEF isolates; vaccine serotype: vaccine-serotype pneumococcus in MEF isolates.
