# Erratum to “Pneumococcal Conjugate Vaccines and Otitis Media: An Appraisal of the Clinical Trials”

**DOI:** 10.1155/2012/590206

**Published:** 2012-09-19

**Authors:** Mark A. Fletcher, Bernard Fritzell

**Affiliations:** Pfizer Specialty Care Business Unit-Vaccines, International Scientific & Clinical Affairs, Pfizer Inc, 75668 Paris Cedex 14, France

Subsequent to the publication of the previous paper entitled “*Pneumococcal Conjugate Vaccines and Otitis Media: An Appraisal of the Clinical Trials,*” scientific errors were discovered in Table 4. Therefore, an erratum was submitted with corrections to the table.

## Figures and Tables

**Table 4:** Vaccine efficacy values in the long-term followup of PCV clinical trials with otitis media as an endpoint [3, 13]. Adapted with permission from Fletcher and Fritzell, 2007 Elsevier Ltd. All rights reserved [7].

	Follow-up clinical trials		
		NCKP [3]	FinOM [13]
Study vaccine		PCV7-CRM	PCV7-CRM
Period		1998-1999	1999–2001
Number		27,754	756
Age		Until aged 3.5 years	Until aged 4-5 years

	Clinical endpoints^*∗*^: vaccine efficacy, % (95% CI)		

Otitis media visits (NCKP) or episodes (FinOM)	PP	8 (5–11)	8 (−2 to 16)
	ITT	7 (5–9)	—
Recurrent otitis media^*∗∗*^			
3/4		10 (7–13)	18 (1–32); 50 (15–71)^†^
5/6		—	—
≥10^‡^		26 (12–38)	—
All tympanostomy-tube placements		23 (−10 to 46)	39 (4–61)^§^; 49 (−7 to 76)^§†^ 44 (19–62)^¶^; 63 (28–81)^¶†^
Rate of AOM-related ambulatory visits		8 (5–10)	—
Rate of antibiotic prescriptions for AOM		6 (4–7)	—

^*∗*^See Table  1 for primary otitis media endpoint, otitis media definition, myringotomy criteria, and source of MEF in each study.

^*∗∗*^Number of episodes in 6 months/number of episodes in 1 year.

^†^In children diagnosed with “chronic otitis media with effusion.”

^‡^Efficacy against 10 or more episodes within 6 months.

^§^Primary analysis set (see Section  3.5.2. FinOM vaccine trial).

^¶^Secondary analysis set (see Section  3.5.2. FinOM vaccine trial).

Reported values are rounded to whole numbers; dash line indicates not reported.

CI: confidence interval; ITT: intent to treat; PP: per-protocol.

